# Relevance of Macrophage Extracellular Traps in *C. albicans* Killing

**DOI:** 10.3389/fimmu.2019.02767

**Published:** 2019-12-04

**Authors:** Ana Loureiro, Célia Pais, Paula Sampaio

**Affiliations:** Centre of Molecular and Environmental Biology (CBMA), Department of Biology, University of Minho, Braga, Portugal

**Keywords:** *Candida albicans*, macrophage extracellular traps, multiplicity of infection, antifungal activity, DNase virulence factor

## Abstract

*Candida albicans* causes systemic life-threatening infections, particularly in immunocompromised individuals, such as patients in intensive care units, patients undergoing chemotherapy, and post-surgical and neutropenic patients. The proliferation of invading *Candida* cells is mainly limited by the action of the human innate immune system, in which phagocytic cells play a fundamental role. This function is, however, limited in neutropenic patients, who rely mainly on the protective immunity mediated by macrophages. Macrophages have been shown to release extracellular DNA fibers, known as macrophage extracellular traps (METs), which can entrap and kill various microbes by a process called ETosis. In this study, we observed that, upon contact with *C. albicans*, macrophages became active in phagocyting and engulfing yeast cells. ETosis was induced in 6% of macrophages within the first 30 min of contact, and this percentage increased with the multiplicity of infection until a plateau was reached. After 2.5 h incubation, the presence of extracellular macrophage DNA was observed in approximately half of the cells. This study suggests that the formation of METs occurs before pyroptosis (first 6–8 h) and macrophage cell death (up to 24 h), and thus, METs could be included in models describing *C. albicans*–macrophage interactions. We also observed that macrophage ETosis and phagocytosis can occur simultaneously and that, in the first hours of infection, both processes are similarly important in controlling the proliferation of yeast cells, this being critical in neutropenic patients. Finally, it can also be concluded that, since *C. albicans* can degrade DNA, the structural component of METs, yeast extracellular DNase activity can be considered as an important virulence factor.

## Introduction

An effective host response against microbial infections requires the coordinated contribution of the innate and adaptive immune systems (1, 2). Macrophages and neutrophils are essential professional phagocytes that are capable of carrying out various roles in the host's innate defense against pathogens (3, 4), and phagocytosis is an essential stage of this defense (5). This process starts with the engulfment of the hostile cell through actin-dependent mechanisms (6) and formation of the phagosome, which then fuses with the lysosome to produce the phagolysosome (3). Inside the phagolysosome, the pH is lowered, and antimicrobial compounds, reactive oxygen species, and reactive nitrogen species are produced to induce microbial cell death (7). Hence, phagocytic cells have a key role in the innate immune response and also in the activation of adaptive immune responses.

Recently, a novel antimicrobial process has been described, the extracellular Traps process (ETs). This involves web-like structures composed of double-stranded DNA, histones, antimicrobial peptides, and proteases, which are ejected by immune cells to entrap microbes in a sticky matrix of extracellular chromatin and microbicidal agents (8, 9). These structures were first described in neutrophils and called “neutrophil extracellular traps” (NETs), and the process of killing has been termed “NETosis” (10). Similar extracellular structures were also reported in macrophages (11), mast cells (12), and eosinophils (13), and this mechanism is now generalized as “ETosis.” Importantly, the extracellular structures arising from different cell types can exhibit unique features, being distinct from those originally described for neutrophils. The released structures may be composed of chromatin, granule proteins such as neutrophil elastase and myeloperoxidase, and histones (14), and exhibit well-characterized antimicrobial properties (14–16). There are also reports demonstrating that, in addition to chromosomal DNA, mitochondrial DNA could also be used by eosinophils (13) and neutrophils (17) to form ETs. The primary function of ETs has been attributed to their antimicrobial effect. However, the overall role of ETs in host defense remains a topic of debate since the mechanisms behind their formation are still unclear. Besides, recent studies have associated ETs with immunological disorders and pathogenesis of certain vascular physiology disorders, including pre-eclampsia (18) and deep-vein thrombosis (19), as well as autoimmune diseases such as rheumatoid arthritis (20).

Formation of extracellular traps (ETs) generally begins with the loss of nucleus organization followed by chromatin decondensation and nuclear membrane disruption. At the same time, cytoplasmatic granular membranes also undergo disruption and leads to the mixing of granular content with the chromatin leaking into the cytoplasm. Finally, the cellular membrane disrupts, and DNA mixed with the granular content is released into the extracellular milieu (21).

*Candida* spp. are common pathogens in hospital-acquired infections (22–25), particularly in immunocompromised individuals, among which are intensive care, post-surgical, and neutropenic patients (26). Indeed, *Candida albicans* is the most frequently isolated human fungal pathogen; it causes systemic life-threatening infections, and despite the currently available antifungal therapies, these infections are associated with high mortality and morbidity rates (27, 28).

*Candida albicans* is known to activate neutrophils to induce NETs development, and these NETs can capture and kill *C. albicans* in both the yeast and hyphal morphologies (15). The released NETs seem to attach to the microbial cell wall, probably through ionic forces, and the protein-containing granules present in the NETs display antimicrobial properties which induce cell death (15). In neutropenic patients, however, the severely reduced neutrophil levels result in reduced antimicrobial effect of NETs. Importantly, *C. albicans* has also been found to induce ET formation in macrophages/monocytes (29, 30) and eosinophils (31), and these may play a protective role in these patients. It has been described that human monocytes release DNA during the initial hours of contact with *C. albicans* and that these ETs have antifungal activity and reduce *C. albicans* growth (29). Murine J774A.1 macrophage-like cells were also found to form ETs, but these were not found to have killing effects on the trapped *C. albicans* (29, 30). In the present study, we show that macrophages exert their antifungicidal activities by phagocytosis and ETosis simultaneously. In our assay, we found that ETosis increases with time and multiplicity of infection (MOI). At a MOI of 25:1, ETosis reached a maximum between 1 and 1.5 h after infection. Interestingly, macrophage cells committed to phagocytosis were not found to undergo ETosis or pyroptosis during the first 4.5 h of interaction. Considering the current model of *C. albicans*–macrophage interaction, these results suggest that METs' formation occurs before pyroptosis (first 6–8 h) and macrophage cell death (up to 24 h). We also observed that the yeast killing efficacy of ETosis and phagocytosis is similar and that *C. albicans* cells can degrade extracellular DNA, which is the main structural component of METs.

## Materials and Methods

### Microbial Strains and Media

*Candida albicans* clinical isolate SC5314 was used. The strain was stored in 30% glycerol at −80°C and, when needed, maintained at 4°C in yeast extract peptone dextrose (YPD) agar medium containing 1% (*w*/*v*) Bacto Peptone, 0.5% (*w*/*v*) yeast extract, 2% (*w*/*v*) glucose, and 2% (*w*/*v*) agar. For *in vitro* assays, the cells were cultured in YPD medium overnight at 26°C and 140 rpm to maintain cells in the yeast form. Cells were counted in a hemocytometer and normalized to appropriate concentrations. In some cases, dead yeast cells were used, which were prepared by boiling for 30 min.

### Macrophages Isolation and Maintenance

Murine macrophage-like cell line J774A.1 was used for most of the experiments. This cell line was maintained at 37°C, in an atmosphere that contained 5% CO_2_, in Dulbecco's modified Eagle's medium (DMEM) supplemented with 10% heat-inactivated fetal bovine serum, 2 mM l-glutamine, 1 mM sodium pyruvate, and 10 mM 4-(2-hydroxyethyl)-1-piperazineethanesulfonic acid (HEPES) buffer. Before use, the adherent cells were gently scraped from the plates, centrifuged at 1,200 rpm for 10 min at 4°C, and diluted in 2 ml DMEM. The trypan blue (Sigma-Aldrich) exclusion assay was used for counting and viability analysis, and a suspension of macrophages was prepared at a concentration of 2.5 × 10^5^ cells/ml.

BALB/c bone-marrow-derived macrophages (BMDMs) and macrophages isolated from the peritoneal cavity after eliciting with 8% casein were also used. For the preparation of BMDM, BALB/c mice were killed and their hind limbs removed, isolating the tibia and femur. DMEM medium was injected into the bones and the resulting medium recovered. After centrifugation at 1,200 rpm for 10 min at 4°C, the cell pellet was suspended in Roswell Park Memorial Institute (RPMI) medium [10 mM HEPES buffer, 0.5 mM 2-β-mercaptoethanol, 50 μg/ml/100 IU/ml streptomycin/penicillin and 10% (*v*/*v*) inactivated fetal bovine serum] supplemented with L929 cell-conditioned medium (LCCM). LCCM was obtained from a fibroblast culture in RPMI for 7 days at 37°C and 5% CO_2_, being the source of macrophage-colony-stimulating factor 1. After overnight incubation, the cells were washed by centrifugation at 1,200 rpm for 10 min at 4°C. The cell suspension was then prepared, seeded in 24-well-tissue culture plates, containing coverslips, and incubated for 4 days. Following incubation, 100 μl LCCM was added to each well, and the cells were incubated a further 2 days. Following these 6 days of total incubation time, the medium was renewed and the cells used in the assays as required. For primary murine peritoneal macrophages, mice were injected in the peritoneal cavity with 0.5 ml of an 8% casein solution in PBS. Seventy-two hours after the casein injection, mice were killed by CO_2_ exposure and 5 ml of PBS injected for peritoneal lavage. The peritoneal fluids were then carefully recovered, avoiding any possible contamination. Cells were normalized to the desired concentrations and then seeded in tissue culture plates containing coverslips for the assays. All procedures involving mice were performed according to the European Convention for the Protection of Vertebrate Animals Used for Experimental and Other Scientific Purposes (ETS 123), the 86/609/EEC directive, and Portuguese rules (DL 129/92).

### *C. albicans*–Macrophage Interaction Assay

The macrophage cell suspensions (1 mL) were seeded into 24-well tissue culture plates, with coverslips in each well, at a cell concentration of 2.5 × 10^5^ cells/mL and incubated overnight at 37°C in 5% CO_2_. Macrophages were then infected with 0.2 mL of the *C. albicans* cell suspension at several MOIs: 5:1, 10:1, 25:1, 50:1, and 100:1 (*C. albicans*–macrophages). After 30 min, 1, and 4 h of infection, cells were examined by light microscopy. For Hemacolor staining following incubation, cells were fixed to the coverslips with formol–ethanol (1:9 *v*/*v*) for 1 min, washed with PBS, and stained with Hemacolor Red solution for 3 min followed by Hemacolor Blue solution for min. After staining, the coverslips were washed in water and observed. For fluorescence microscopy, following incubation, cells were fixed with 4% paraformaldehyde, stained with Sytox Green (Molecular Probes) at a final concentration of 5 μM, and observed with a Leica DM5000B fluorescence microscope. A minimum of 250 cells were counted in each slide, from a total of 6 different slides from 3 independent experiments.

### MET Induction Through Non-cellular Stimuli: DNA Quantification

Macrophages were seeded overnight at a cell concentration of 2.5 × 10^4^ cells (200 μl) in 96-well-plates before being incubated with lipopolysaccharide (LPS), phorbol-12-myristate-13-acetate (PMA), interferon-gamma (IFN-γ), *N*-acetylglucosamine, and mannan from *Saccharomyces cerevisiae* for 1 h. The concentrations tested ranged from 10 to 1,000 ng/ml LPS, 6.25–200 nM PMA, 6.25–200 ng/ml IFN-γ, 12.5–400 μg/ml *N*-acetylglucosamine, and 31.25–1,000 μg/ml yeast mannan. The cells were then washed with PBS, and Sytox Green (Molecular Probes) was added at a final concentration of 5 μM to detect extracellular DNA. Controls, with macrophages only, were also carried out. The intensity of fluorescence, as a direct quantification of the amount of DNA released, was determined with a Xenogen Vivo Vision IVIS 200 imaging System (Xenogen Corporation, Hopkinton) with excitation at 485 nm and emission at 527 nm. To test double stimuli, macrophages were prepared as described previously and stimulated with LPS at concentrations of 1 μg/ml, 100, and 10 ng/ml for 20 min. The cells were then washed with PBS and incubated with *C. albicans* in PBS at a MOI of 25:1. After 1 h incubation, Sytox Green (Molecular Probes) was added and the amount of DNA released determined as described previously.

### Identification of DNA as a Major Structural Component of METs

J774A.1 macrophage-like cells were infected with *C. albicans* at a MOI of 25:1 and incubated at 37°C in 5% CO_2_ for 1 h. Cells were then fixed to coverslips, stained with Sytox Green, and treated with the mouse monoclonal antihistone H2A-H2B-DNA complex antibody (gift of Dr. Volker Brinkmann, Max Planck Institute for Infection Biology, Berlin) according to Fuchs et al. (8). The preparations were observed using a Leica DM5000B microscope and Olympus FluoView FV1000 confocal microscope. To further confirm that DNA is the main structural component of METs, macrophages were incubated with *C. albicans*, as described previously, but in the presence of DNase (100 U/ml) and protease (trypsin, 0.25%) at the time of infection. After fixation, cells were stained with Sytox Green or with the Hemacolor staining protocol, as previously described.

### Evaluation of *C. albicans* Secreted DNase Activity

J774A.1 macrophage-like cells were incubated with live and heat-killed *C. albicans* cells at a MOI of 25:1 at 37°C in 5% CO_2_ for 1 h and the supernatants collected. One arbitrary PCR DNA fragment was incubated with these supernatants for 30 min and then subjected to 1% agarose gel electrophoresis in Tris–borate–EDTA buffer. The gels were stained with ethidium bromide and photographed. Image J software (version 1.51) was used for the analysis of the intensity of DNA fragments in the agarose gel.

### Antimicrobial Activity of Macrophage Extracellular Structures

To determine the antimicrobial effect of METs, the survival of *C. albicans* in contact with macrophages was quantified. Macrophages from the cell line J774A.1 were prepared and seeded in 24-well-tissue culture plates as previously described. After adhesion, the medium was removed and replaced with DMEM medium, or with DMEM supplemented with the inhibitor cytochalasin D at a concentration of 0.25 μg/ml. Plates were then reincubated for 20 min and the medium removed before adding the *C. albicans* suspension simultaneously with treatment with DNase (100 U/ml). The cells were further incubated at 37°C, 5% CO_2_ for 1 h and 800 μl water with 10% saponin added to each well to facilitate removal of adhered *C. albicans* cells from the wells. To confirm cell removal, up and down pipetting was performed several times, and the plates were examined under a binocular magnifier. The obtained suspension was diluted and seeded on YPD agar plates and incubated overnight at 37°C before counting to determine the number of viable *C. albicans* cells as colony-forming units.

### Statistical Analyses

Statistical analyses were performed using Graph Pad Prism (version 7), and significance was determined using two-way ANOVA with Tukey's multiple comparison test. All tests were performed with a confidence level of 95%. All experiments were done in duplicate, and results were obtained from three independent experimental assays.

## Results

### Macrophage Extracellular Fiber Production in Response to *C. albicans* Cells

Macrophages are dynamic cells, distributed in various tissues, which play important roles in immune processes and whose functions are vital to host immune defense and tissue homeostasis. These professional phagocytes play key roles in initiating inflammation and orchestrating its resolution. Their main conventional antimicrobial ability is to eliminate microbial pathogens through phagocytosis, using a combination of oxidative and non-oxidative microbicidal mechanisms.

**Figure 1:**
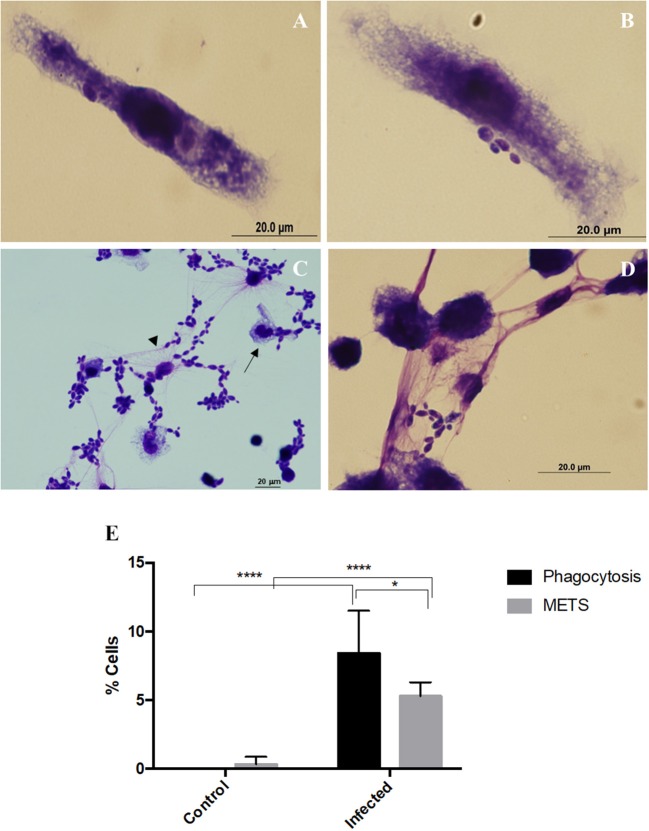
Extracellular fibers observed after macrophages–*C. albicans* interaction *in vitro*. Hemacolor staining images of the structures observed following *in vitro* interaction of *C. albicans* and J774A.1 macrophage-like cells for 30 min. Macrophages exhibiting typical phagocytic structures **(A,B)**. Extracellular structures resembling macrophage extracellular traps (METs) **(C,D)**. The arrowhead points to extracellular structures that entrap *C. albicans* cells and the arrow to phagocytosis. Quantification of macrophages exhibiting phagocytosis and METs **(E)** after infection with *C. albicans*. *P < 0.05 and ****P < 0.0001 by the Tukey's multiple comparisons test.

Incubation of macrophages with *C. albicans* was performed and cells fixed and stained with Hemacolor (Figure 1). Different structures were observed in this assay, namely elongated macrophages with yeast cells attached or internalized (Figures 1A,B), and macrophages interconnected to each other by extracellular structures that resembled ETs trapping the yeast cells (Figures 1C,D). After 30 min incubation with *C. albicans*, 8.4% of macrophages were engaged in phagocytosis and 5.3% formed extracellular structures (Figure 1E). In a more detailed analysis, it was possible to observe that both types of morphologies were present simultaneously (Figure 1C, arrows).

### Macrophage Release of Extracellular DNA

To determine whether these extracellular structures consisted of DNA, the assay was repeated with not only the macrophage-like cells from J774A.1 but also BMDM and primary murine peritoneal macrophages. At the end of the incubation period, the cells were fixed with 4% paraformaldehyde, stained with Sytox Green and observed under a fluorescence microscope (Figure 2.1A–F). A quantification of cells with METs was also performed (Figure 2.1G). Results show that the extracellular structures released by J774A.1 macrophage-like cells (Figure 2.1D), by a derived primary culture (BMDM) (Figure 2.1F), and by primary murine peritoneal macrophages (Figure 2.1E) consisted of DNA, since they were stained by the DNA staining dye Sytox Green. As expected, uninfected macrophages showed no or only residual ET structures (Figure 2.1A–C).

**Figure 2:**
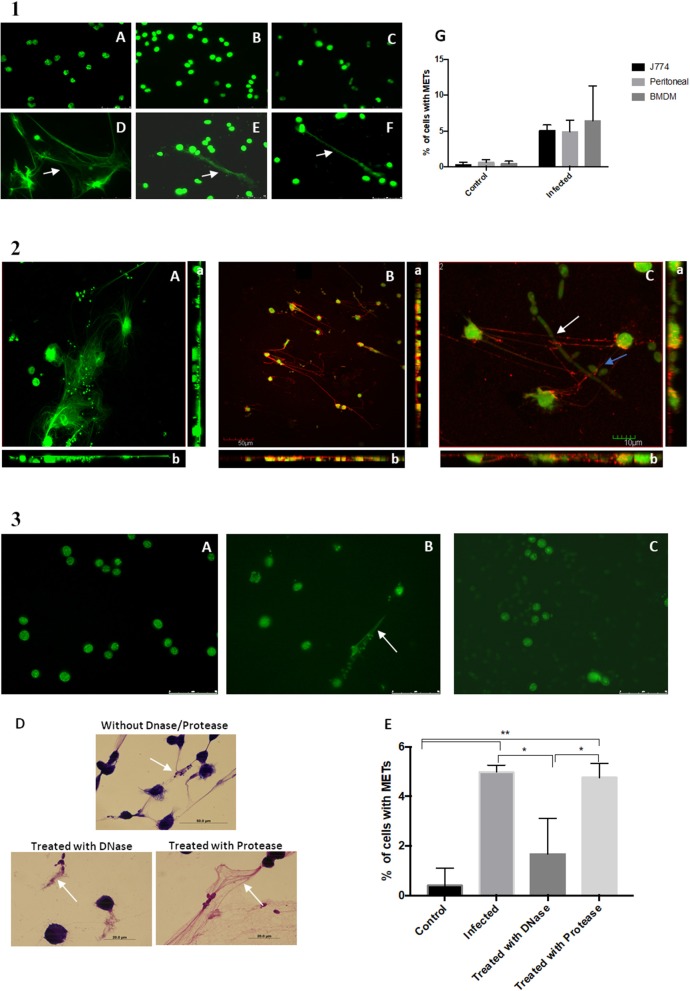
DNA release following *in vitro* incubation of macrophages with *C. albicans* cells. **(1)** Fluorescence images of macrophage cells incubated with *C. albicans* for 30 min and stained with Sytox Green. Control of J774A.1 macrophage-like cells (A) and J774A.1 macrophage-like cells incubated with *C. albicans* (D). Control cells of primary murine peritoneal macrophages (B) and the same cells incubated with *C. albicans* (E). Bone-marrow-derived macrophages (BMDM) from BALB/c mice control cells (C) and BMDM infected with *C. albicans* (F). Quantification of macrophages exhibiting macrophage extracellular traps (METs) (G) after infection with *C. albicans*. **(2)** Fluorescence images of J774A.1 macrophage-like cells entrapping *C. albicans* stained with Sytox Green and anti-H2A-H2B-DNA complex antibody (B); macrophages in more detail entrapping *C. albicans* yeast cells (blue arrow) and hyphae (white arrow) (C); J774A.1 macrophage-like cells incubated with *C. albicans* cells and stained only with the secondary antibody and Sytox Green (A). Rotation around the *y*-axis (a); rotation around the *x*-axis (b). Red corresponds to the labeling with anti-H2A-H2B-DNA complex antibody; green corresponds to the marking with Sytox Green. **(3)** Fluorescence images of control J774A.1 macrophage-like cells (A), macrophages infected with *C. albicans* (B), and macrophages infected with *C. albicans* and treated with DNase (C) followed by Sytox Green staining. Hemacolor images (D) of macrophages infected with *C. albicans* and treated with trypsin, with DNase, or not treated. Quantification of macrophages exhibiting METs (E) after infection with *C. albicans* in the presence and absence of treatments. *P < 0.05 and **P < 0.01 by the Tukey's multiple comparisons test.

Confocal microscopy confirmed that these ETs could be readily visualized with an antibody against histone–DNA complexes, which allowed us to confirm that DNA and histones are the main components of these structures (Figure 2.2). These results are in agreement with the previous observed phenotypes of NETs (14). In addition, it was possible to observe that the METs could entrap both yeast and hyphae *C. albicans* structures (Figure 2.2C and Videos S1, S2).

Induction of the METs was performed in the presence of DNase and protease (trypsin) and resulted in a significant difference in the percentage of ETs being produced (Figure 2.3E). Fluorescent (Figure 2.3C) and Hemacolor images (Figure 2.3D) showed no clear formation or destruction of METs in the presence of DNase, in contrast to induction of ETs in the absence of DNase (Figure 2.3B) or the presence of protease. These results confirmed that the ETs contain DNA and are thus susceptible to DNase degradation.

### ET Formation Increases With Increasing Multiplicity of Infection

**Figure 3:**
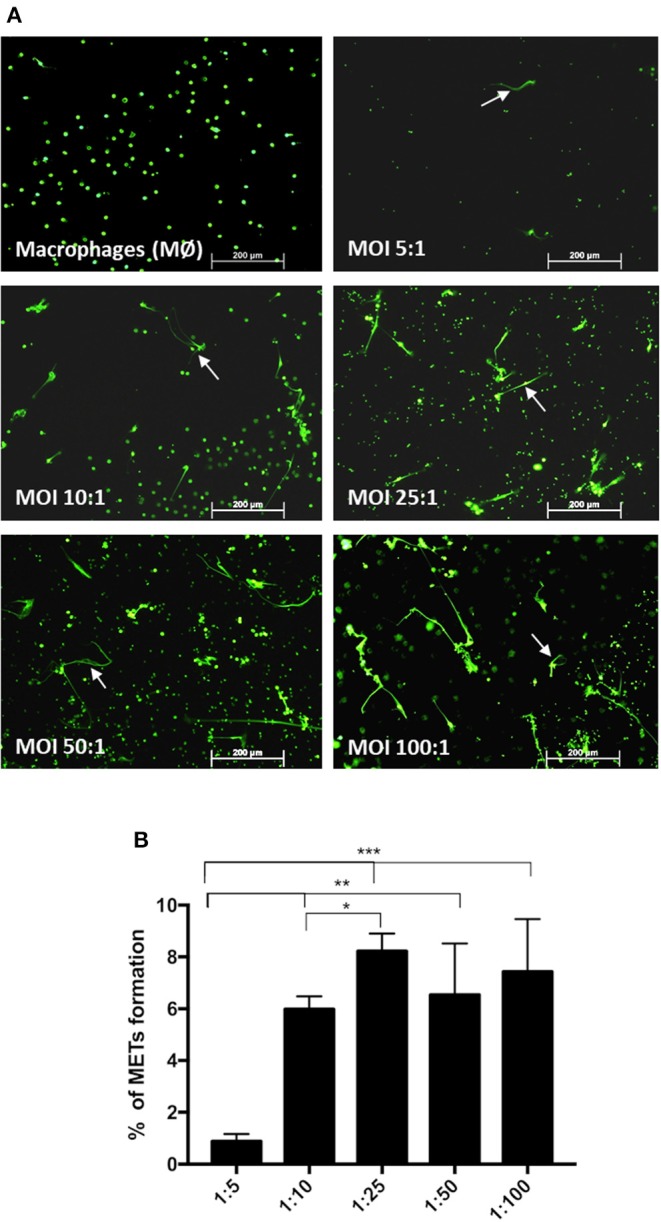
Frequency of macrophage extracellular trap (MET) formation with multiplicity of infection. Fluorescence images (arrows point to METs) **(A)** and quantification of the formation of METs **(B)** by J774A.1 macrophage-like cells incubated with *C. albicans* at different MOI (5:1, 10:1, 25:1, 50:1, and 100:1; *C. albicans*–macrophages) for 30 min and stained with Sytox Green. *P < 0.05, **P < 0.01, and ***P < 0.001 by the Tukey's multiple comparisons test.

Incubation of macrophages with *C. albicans* at different MOIs of 5:1, 10:1, 25:1, 50:1, and 100:1 (*C. albicans*–macrophages) showed that increasing the number of *C. albicans* cells induced an increase in the percentage of METs produced (Figure 3) until an MOI of 25:1. Above an MOI of 25:1, no significant differences were observed in the frequency of ETs, regardless of the number of yeast cells present.

### Macrophages Involved in Phagocytosis Do Not Undergo ETosis

The kinetics of MET formation was evaluated in primary murine peritoneal macrophages after infection with *C. albicans* cells and evaluated over 2.5 h by real-time imaging in the presence of Sytox Green (Video S3). Figure 4.1A shows representative images taken at different time points. In Figure 4.1B, we can observe that the percentage MET production increased progressively with time. Statistical analysis indicated that nuclear staining was significantly different from baseline after only 78 min incubation (30.7% METs) but that between 87 min (34.8% METs) and 150 min (56.0% METs), there were no significant differences. This analysis also showed that MET morphology is not uniform (Figure 4.2A); cells may present a diffuse extracellular chromatin morphology (puffball-like) (Figure 4.2A, asterisk), a spread extracellular chromatin morphology (comet-like) (Figure 4.2A, number sign), or an extended strands morphology that interlinks across cells (Figure 1). These morphologies were already described in other studies (32).

**Figure 4:**
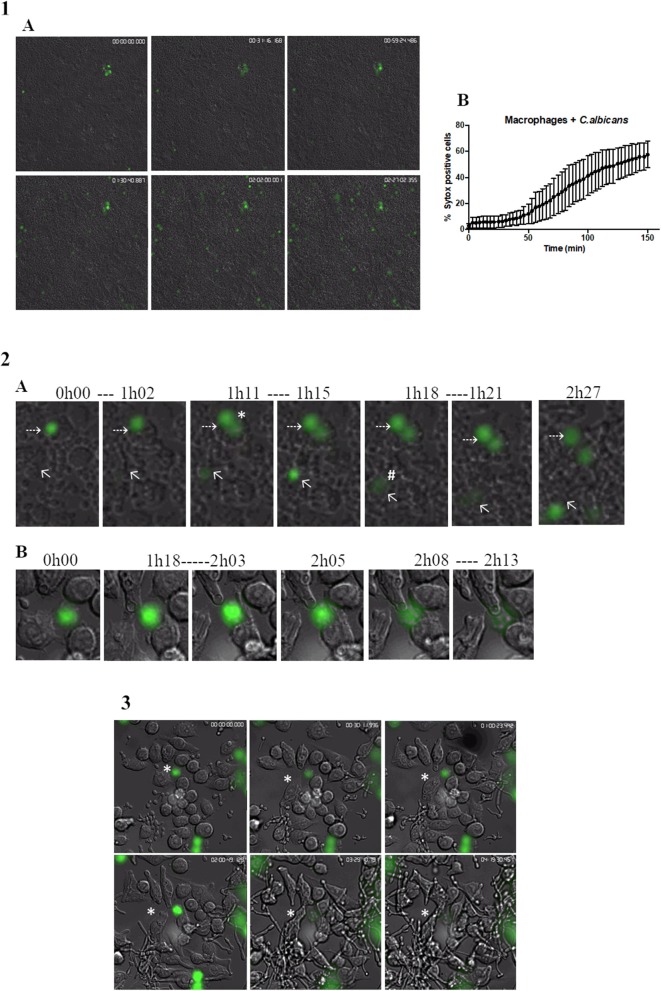
Live cell imaging of macrophages incubated with *C. albicans*. (**1**A) Representative images of primary murine peritoneal macrophages incubated with *C. albicans* and Sytox Green, images taken at selected time points during the 2.5-h incubation time (images taken with a 10× objective). (**1**B) Quantification of Sytox-positive cells over time. Images representative of nine replicates. (**2**A) Representative images of macrophage cells observed in (**1**A) where we can discern different MET morphologies. Asterisk represents a diffused extracellular chromatin and number sign a spread extracellular chromatin. (**2**B) Representative images of the kinetics of MET formation (images from **3**). Dashed arrows points to a cell that remained at the puffball-like morphology over the time studied. Solid arrows points to a cell that progressed to comet-like morphology. **(3)** Representative images of primary murine peritoneal macrophages incubated with *C. albicans* and Sytox Green, images taken at selected time points during the 4.5-h incubation time (images taken with a 40× objective). Images representative of six replicates.

The kinetics of the formation of METs is also variable. Some cells were found to present a puffball-like morphology (Figure 4.2A, dashed arrow) and remained as such during the 2.5-h analysis time, while others were Sytox Green positive and progressed rapidly (16 min total) to a comet-like morphology (Figure 4.2A, solid arrow). Others still, as represented by the cell in Figure 4.2B, were already in a diffuse morphology at the beginning of the video analysis but progressed to a comet-like morphology after 2 h and 8 min.

*Candida albicans* cells can form filaments inside macrophages that could disrupt the macrophage cell, and thus, the Sytox Green signal observed could be due to this disruption and not to MET release. Therefore, a closer look was taken at cells undergoing phagocytosis overtime (Figure 4.3 and Video S4). Results show that *C. albicans* cells are efficiently phagocytosed by macrophages; however, once phagocytosed and contained within a phagosome, the yeast can still form hyphae, leading to the stretching of phagocyte membranes. Nevertheless, during the analysis of 4.5-h duration, these macrophages did not stain with Sytox Green, which indicates that they did not induce METs or undergo pyroptosis. Considering the current model of *C. albicans*–macrophage interaction, these results suggest that MET formation is a mechanism that occurs before pyroptosis (first 6–8 h) and macrophage cell death (up to 24 h).

### Induction of METs by Non-cellular Stimuli

**Figure 5:**
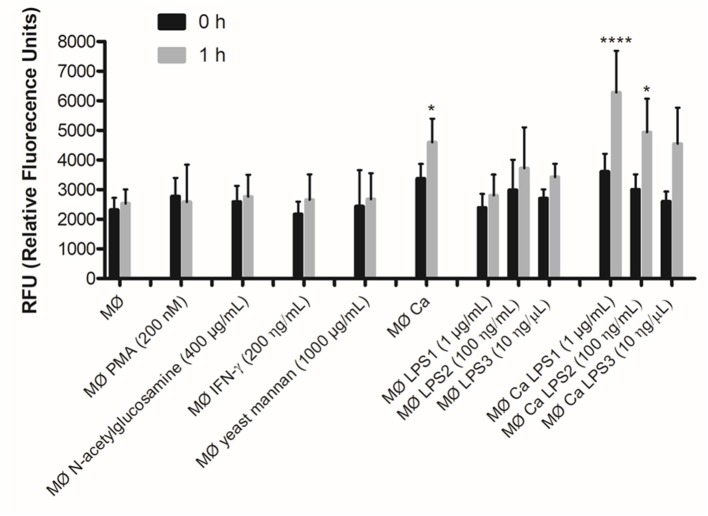
DNA release in response to non-cellular stimuli and *C. albicans*. J774A.1 macrophage-like cells stimulated with 200 nM phorbol-12-myristate-13-acetate (PMA), 400 μg/ml *N*-acetylglucosamine, 200 ng/ml interferon-gamma (IFN-γ), 1,000 μg/ml yeast mannan, 1 μg/ml [lipopolysaccharide 1 (LPS1)], 100 ng/ml (LPS2), or 10 ng/ml (LPS3) LPS, and incubated with *C. albicans* alone or with yeast and LPS simultaneously. Extracellular DNA was quantified by fluorescence analysis. *P < 0.05 and ****P < 0.0001 by the Tukey's multiple comparisons test, in comparison with macrophages alone.

The yeast cell wall is composed of an outer layer of glycosylated proteins with mannosyl residues and an inner layer of β-glucans and chitin (33). Taking into account that *C. albicans* induced the formation of METs, we tested whether, individually, mannans (mannose polymer) or *N*-acetylglucosamine (the monomeric unit of chitin) could induce the formation of METs. In addition, as PMA and LPS stimuli have been shown to activate neutrophils to release NETs (14), and as IFN-γ is an important cytokine in macrophage activation, we also tested these stimuli in macrophages. The results showed that, except for LPS, the tested stimuli did not significantly enhance the formation of METs, and LPS only induced a slight increase after 1-h incubation (Figure 5). Therefore, we decided to quantify the formation of METs when macrophages were incubated with LPS and *C. albicans* (Figure 5). Results indicate significant differences (*P* < 0.05) in the formation of METs for macrophages incubated with *C. albicans* as compared with macrophages alone. When *C. albicans* and LPS were combined, a significant difference was also observed in comparison with macrophages alone: *C. albicans* with 1 μg/ml LPS *P* < 0.0001 and *C. albicans* with 100 ng/ml LPS *P* < 0.05. However, since no significant differences were observed with LPS incubations alone, these differences with *C. albicans* and LPS were mainly due to the presence of the yeast. Thus, even combining *C. albicans* with LPS, the release of METs is mainly due to the presence of *C. albicans* and not to LPS.

### Dead and Live *C. albicans* Cells Are Able to Induce METs

**Figure 6:**
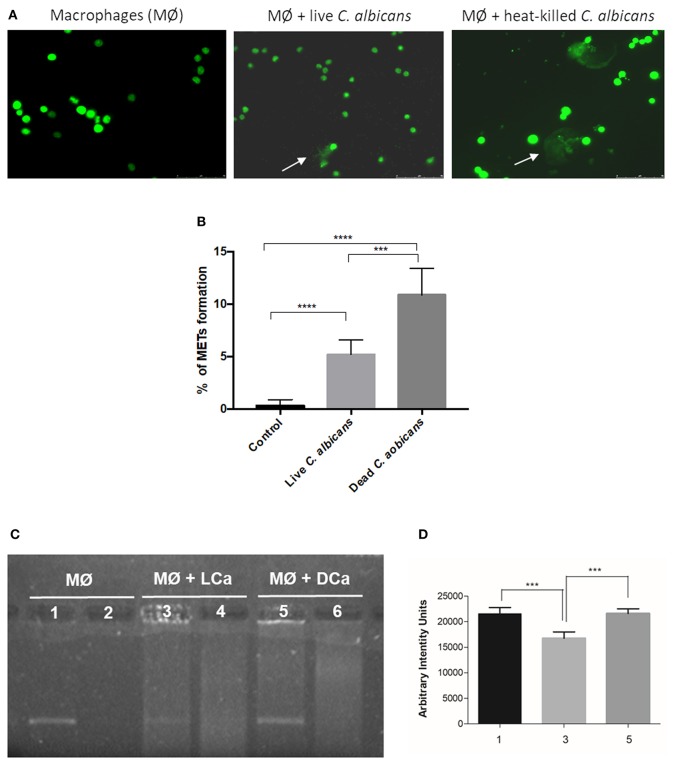
MET formation by live and heat-killed *C. albicans* cells. Representative fluorescence images of J774A.1 macrophage-like cells infected with live cells and heat-killed *C. albicans* **(A)** and quantification of macrophage extracellular trap (MET) formation **(B)**. Arrows indicate the presence of macrophage extracellular traps (ETs). Evaluation of DNase activity in the supernatants of incubations: agarose gel **(C)** and intensity quantification **(D)** of a DNA fragment incubated with supernatant of macrophages alone (1), supernatant of macrophages infected with live *C. albicans* cells (3), and supernatant of macrophages infected with heat-killed *C. albicans* cells (5). Negative controls, without DNA addition, of (2) only the supernatant of macrophages, (4) the supernatant of macrophages infected with live *C. albicans*, and (6) the supernatant of macrophages infected with heat-killed *C. albicans*. ***P < 0.001 and ****P < 0.0001 by the Tukey's multiple comparisons test.

Since the different components of the *C. albicans* cell wall were not able to significantly induce the formation of METs, we incubated macrophages with *C. albicans* live and heat-killed cells at the same MOI (25:1) for 1 h (Figure 6). Results indicate that dead *C. albicans* cells are also able to induce macrophage extracellular structures (Figure 6A) and even showed a higher amount of these structures (10.9% ± 2.5) when compared with macrophages incubated with live yeast cells (5.2% ± 1.4) (Figure 6B). One possible explanation for this difference is that the live *C. albicans* cells can secrete compounds, such as DNases, which may degrade DNA, the main component of METs. To determine if *C. albicans* secretes compounds with the ability to degrade DNA, an arbitrary DNA fragment was incubated with the supernatant of macrophages infected with live and heat-killed *C. albicans* cells (Figure 6C). Agarose gel electrophoresis analysis indicated ~25% degradation of the DNA fragment upon incubation with the supernatant of macrophages stimulated with live *C. albicans* cells (Figure 6D). In contrast, the DNA fragment incubated with the supernatant of macrophages stimulated with dead *C. albicans* cells and with the control (uninfected macrophages) was unaltered. These results suggest that *C. albicans* cells can secret compounds that can degrade the ETs and thereby lead to the observed lower MET levels.

### Macrophage Extracellular Structures Trap and Kill *C. albicans* Cells

**Figure 7:**
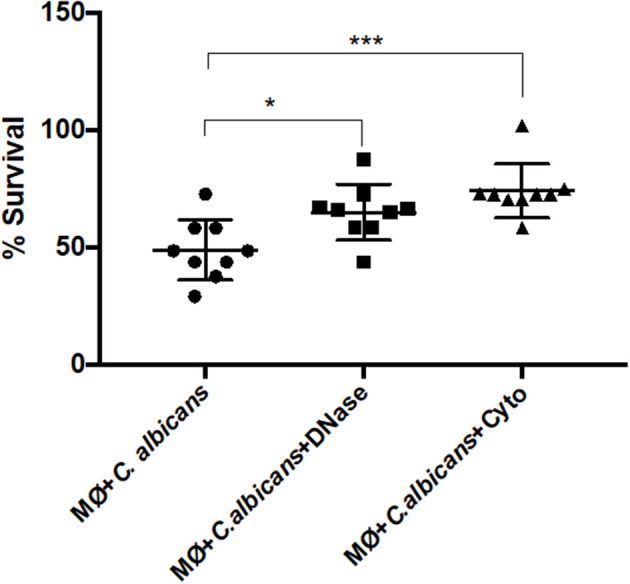
*C. albicans* killing by macrophage extracellular traps (METs). *C. albicans* survival (%) when incubated with J774A.1 macrophage-like cells without any treatment, or with treatment with DNase and/or cytochalasin D. Samples treated with cytochalasin D and DNase I simultaneously were set as the 100% survival control group. *P < 0.05 and ***P < 0.001 by the Tukey's multiple comparisons test.

To test if the extracellular structures can kill the entrapped *C. albicans* cells, we infected J774A.1 macrophage-like cells with the yeast and quantified the percent survival by CFU counting. The percentage of *C. albicans* survival was determined either in the presence of cytochalasin D, which inhibits intracellular death, or in the presence of DNase I, inhibiting extracellular death (Figure 7). In addition, samples of macrophages incubated with *C. albicans* in the presence of both cytochalasin D and DNase I were used as the 100% survival control (34).

After 1-h incubation with macrophages, only 48.88% ± 12.8 of the *C. albicans* cells survived macrophage antimicrobial action. However, with the administration of DNase, the survival percentage increased to 64.96 ± 11.8 (*P* = 0.0242), which indicates that METs contribute to *C. albicans* killing. The same observation was made following cytochalasin D treatment; here, the survival was 74.10% ± 11.5, being significantly different to macrophages incubated with *C. albicans* without any treatment (*P* = 0.0005) and demonstrating that phagocytosis, as expected, is important in the battle against *C. albicans*. Furthermore, although the difference in the survival percentage between the two different treatments is ~10%, this difference is not statistically significant and suggests that both phagocytosis and METs are similarly important in *C. albicans* killing. As a control, *C. albicans* growth in the presence of cytochalasin D and DNase was also evaluated in the same conditions, and a viability of about 93% ± 1.4 was observed, confirming that these treatments do not affect *C. albicans* growth. Thus, it can be concluded from these experiments that METs have an antimicrobial efficacy similar to that of phagocytosis.

## Discussion

Human fungal pathogens causing invasive infections are responsible for around one and a half million deaths every year (35). *Candida* spp. are common pathogens and are the fourth most frequent cause of nosocomial bloodstream infections, with *C. albicans* being particularly common. This human opportunistic pathogenic fungus often causes systemic life-threatening infections in immunocompromised individuals. Despite currently available antifungal therapies, *C. albicans*-associated mortality and morbidity remain high, and more than 50% of infected patients die due to systemic candidemia (27, 28).

Invading *Candida* cells are immediately attacked by the human innate immune system, which involves activation of the complement system, generation of antimicrobial peptides, and action of phagocytic immune cells. The important contribution of phagocytes to the innate immune control of infections is based on phagocytosis, but more recently, ETosis has also been identified. Several studies have indicated that the formation of ETs is an effective mechanism in combating invading pathogens (36). Macrophage phagocytes play key roles in host immune defense and tissue homeostasis, and, in addition to its phagocytic classical function, recent investigations have shown that macrophages are capable of also producing extracellular traps (ETs) that contribute to their antimicrobial function (8, 9, 11). Nevertheless, in macrophages, this contribution is still poorly understood.

In this study, we observed that *C. albicans* induce macrophage ETs formation in murine J774A.1 macrophage-like cells, peritoneal macrophages, and BMDM. Morphology analysis showed macrophages interconnected with each other, trapping the yeast cells within extracellular structures. These interconnected fibrous structures, which were formed between several macrophages and *Candida* cells, have also been described for human monocytes, forming cluster-like structures where groups of yeast cells are interlaced (29). In our assay, the frequency of cells undergoing ETosis ranged from 6% after 30 min incubation to approximately half of the cells after 2.5 h. The values observed are within the ranges reported in the literature, which ranged from ~10 to 50% depending on the time of incubation (11, 29, 30, 32, 37, 38). Characterization of these extracellular structures, staining with Sytox Green, incubation with anti-H2A-H2B-DNA complex antibody, and treatment with DNase confirmed that their main structural component is DNA, as expected (36). Treatment with DNase simultaneous to *C. albicans* incubation did not prevent formation of METs, but the extracellular structures appeared degraded, and the percentage of cells with clear MET formation was reduced. This is in agreement with previously reported observations for bovine macrophages that indicated that METs are susceptible to DNase degradation (11, 29, 30, 37). In the present study, we also discerned that increasing the MOI increased the amount of METs observed, but only until a MOI of 25:1, after which the formation of METs did not increase, regardless of the number of yeast cells present. ETs have been proposed to restrict microbes to the site of infection, thus preventing systemic spreading, which is particularly important for controlling invasive infections by fungal pathogens (39, 40). Since macrophages are tissue cells, being one of the first innate immune cells to encounter invading pathogens, the intertwisted METs, interconnected with each other and trapping the yeast cells, will play an important role in controlling the spread of *C. albicans* cells.

Real-time imaging revealed that, upon contact with *C. albicans*, macrophages rapidly engulfed yeast cells. The percentage of Sytox Green positive cells increased with time, reaching a significant difference from baseline after 78 min incubation, with 30.7% positive cells. Thereafter, the number of Sytox Green positive cells continued to increase until 150 min incubation, reaching 56.0%. The pattern of MET formation was observed to be non-uniform; we observed cells that presented a puffball-like morphology, while other cells presented a comet-like morphology, remaining as such for 2.5 h. We also observed cells that initially showed a puffball-like morphology but later presented a comet-like morphology. These results are in agreement with those previously reported for vertebrate (41) and invertebrate phagocytes (32). In addition, the time frame of MET formation is also in agreement with that previously described for human monocytes; that being that, upon microbial contact, DNA was released within 2–4 h, but in a few cases, the release of extracellular DNA was detected even earlier, after 20–40 min (29).

In this study, during the real-time analysis, macrophages involved in phagocytosis of *C. albicans* cells did not stain with Sytox Green even after 4.5 h incubation. It is well-known that, once phagocytosed, *C. albicans* cells contained within the phagosome can form hyphae, which can lead to stretching of the phagocyte membranes and eventually membrane piercing and killing of the macrophages (42, 43). These studies show that macrophage killing by *C. albicans* occurs in two distinct phases: phase 1 (first 6–8 h) and phase 2 (8–10 h to 18–24 h post-phagocytosis). The early phase is associated with pyroptosis, a proinflammatory macrophage death, whereas the latter phase depends on robust hyphal formation and is independent of pyroptosis. Our study was performed within the first 4.5 h of *C. albicans* interaction with phagocytes and thus was before the time frame described for pyroptosis. Since macrophages involved in phagocytosis did not become Sytox Green positive during the analysis, DNA release by pyroptosis may not yet be relevant in these initial hours of interaction. This result is in agreement with studies of human monocyte ETs, which indicated that ETosis was an early coordinated process occurring upon contact with *C. albicans*, and different from pyroptosis (29).

In contrast to NETs, METs are not induced by LPS or PMA, not even when LPS was incubated with macrophages previous to *C. albicans*, which indicates that no synergism occurs in MET formation. In addition, neither mannans nor *N*-acetylglucosamine, two components of the yeast cell wall, were able to induce the formation of METs. However, as reported for NETs, heat-killed yeast cells were able to induce METs and in higher amounts than live yeast cells. This suggests that METs, in contrast with NETs, may be induced specifically by microbial cells, alive or dead, rather than by microbial components.

Microorganisms are known to have several strategies to evade human immune attack (44). The most prominent *C. albicans* evasion mechanism is the morphological switch to hyphal growth, which can pierce through human phagocyte membranes, killing the cell (42). However, it has also been described that different *C. albicans* isolates can present extracellular DNase activity (45). We observed that the live yeast cells that we used secreted components with DNase activity that degraded an arbitrary DNA fragment. This is in agreement with that observed for NETs, where *C. albicans* cells were able to degrade DNA in the NETs, particularly strain SC5314, the same strain used in our study (46). Since this extracellular DNase activity is strain dependent, this activity may be considered a virulence factor facilitating yeast escape from ET killing by degradation of ETs.

Previous studies have shown that ETs from neutrophils, eosinophils, and mast cells kill microbes, including *Candida* cells (14–16). In this study, we observed that after 1 h incubation, macrophages were able to kill ~50% of the yeast cells, but when DNase was added, only 35% of the yeast cells were killed, indicating that METs contribute to *C. albicans* killing. However, in the presence of cytochalasin D, the percentage of yeast killing was just 26%, confirming the importance of phagocytosis in killing *C. albicans*. Although there are differences in the percentages of yeast killing by the different treatments, results indicate that both phagocytosis and ETosis play a role in the *C. albicans* killing by macrophages. This result is similar to that reported for METs, which showed that human monocytes have antifungal activity (29), but contrary to that reported by Liu et al. (30) for macrophages. This difference may be due to the multiplicity of infection used by Liu et al., which, according to our results, induces only low amounts of METs.

In conclusion, our study demonstrates that macrophages form ETs when exposed to *C. albicans*, and these can entrap and kill the fungal cells. METs seem to be induced by whole *Candida* cells as opposed to by their cell wall components, and moreover, live yeast cells have the capacity to counterattack METs by degrading its main structural component, DNA. Considering the current model of *C. albicans*–macrophage interaction, it seems that the formation METs is a mechanism that occurs prior to pyroptosis (first 6–8 h) and macrophage cell death (up to 24 h) and could be included in this model.

## Data Availability Statement

All datasets generated for this study are included in the article/Supplementary Material.

## Author Contributions

AL performed the experiments, analyzed, interpreted data, and wrote the manuscript. CP supervised the experiments and interpreted data. PS supervised the experiments, analyzed, interpreted data, and assisted in writing the manuscript.

### Conflict of Interest

The authors declare that the research was conducted in the absence of any commercial or financial relationships that could be construed as a potential conflict of interest. The handling editor declared a shared affiliation, though no other collaboration, with all the authors AL, CP, and PS.

## Supplementary Material

Video S1 3D Animation-x rotation of immunofluorescence microscopy.

Video S2 3D Animation-y rotation of immunofluorescence microscopy.

Video S3 Real-time imaging of *C. albicans*/macrophage interaction (amplification 100×).

Video S4 Real-time imaging of *C. albicans*/macrophage interaction (amplification 400×).
